# Differentiation of Urothelial Carcinoma and Normal Bladder Tissues by Means of Fiber-Based ATR IR Spectroscopy

**DOI:** 10.3390/cancers15020499

**Published:** 2023-01-13

**Authors:** Rimantė Bandzevičiūtė, Gediminas Platkevičius, Justinas Čeponkus, Arūnas Želvys, Albertas Čekauskas, Valdas Šablinskas

**Affiliations:** 1 Institute of Chemical Physics, Faculty of Physics, Vilnius University, Saulėtekio av. 3, LT-10257 Vilnius, Lithuania; 2 Institute of Clinical Medicine, Faculty of Medicine, Vilnius University, M. K. Čiurlionio g. 21/27, LT-03101 Vilnius, Lithuania

**Keywords:** bladder cancer, urothelial carcinoma, fiber-based ATR IR spectroscopy, molecular markers

## Abstract

**Simple Summary:**

Non-muscle-invasive bladder cancer (NMIBC) is a complicated locally treated disease with high recurrence rates and high risk of progression to muscle invasive disease. Current standard diagnostic approach based on white light cystoscopy (WLC) is characterized by high false negative rate, excessive invasion of the bladder and high economic burden. Hereby we present the fiber-based attenuated total reflection infrared (ATR IR) spectroscopy study of healthy and cancerous tissue samples taken from 54 patients to delineate normal and tumorous human bladder tissues under ex vivo conditions. Investigation of the tissue samples immediately after surgical operation allows to examine samples in their native conditions and establish their initial structure and chemical composition avoiding sample degradation. Our study reveals that fiber-based ATR IR spectroscopy could be an addition to current endoscopic approach with increased diagnostic accuracy and with some potential to be applied in vivo.

**Abstract:**

Surgical treatment is widely applied curative approach for bladder cancer. White light cystoscopy (WLC) is currently used for intraoperative diagnostics of malignant lesions but has relatively high false-negative rate. Here we represent an application of label free fiber-based attenuated total reflection infrared spectroscopy (ATR IR) for freshly resected human bladder tissue examination for 54 patients. Defined molecular spectral markers allow to identify normal and urothelial carcinoma tissues. While methods of statistical analysis (Hierarchical cluster analysis (HCA) and Principal component analysis (PCA)) used for spectral data treatment allow to discriminate tissue types with 91% sensitivity and 96–98% specificity. In the present study the described method was applied for tissue examination under ex vivo conditions. However, after method validation the equipment could be translated from laboratory studies to in situ or even in vivo studies in operating room.

## 1. Introduction

Bladder cancer (BC) is the tenth most diagnosed cancer worldwide, with approximately 573,000 new cases and 213,000 deaths annually. It is approximately four times more common in men than in women, with respective incidence and mortality rates of 9.5 and 3.3 per 100,000 among men [1]. Non-muscle-invasive bladder cancer (NMIBC) locally treated and highly recurrent disease which involves only the urothelium or lamina propria without invasion to the detrusor muscle, accounts for about 80% of all bladder cancers [2].

The 5-year recurrence and progression rates depend on clinical and pathological factors and varies from 31% to 78% and from 0.8% to 45%, respectively [3]. Because of the high recurrence rate and complexity of the invasive diagnostic procedures, NMIBC is associated with a high humanistic and economic burden to maintain per patient over his lifetime [4].

White light cystoscopy (WLC), endoscopy of the urinary bladder via urethra, combined with transurethral resection of the bladder (TURB) procedure and subsequent pathology examination of the specimens is the mainstay of diagnosis in BC. This diagnostic approach enables to access the local staging and grading of the disease to determine the optimal patient management tactic [5]. However, WLC has a negative predictive value (NPV) of 56% [6], which could be increased up to 86% in combination with urinary cytology examination [7].

Furthermore, it requires surgical resection of the bladder wall. Complication rates are approximately 4–6% of which urinary tract infections and significant hematuria are the most common [8]. Some of these resections are not necessary, as we know from the literature that positive predictive value (PPV) of WLC alone is only 64% [6]. As a result, novel technologies are under development to improve lesion detection, diagnostic accuracy, and prognosis, when having minimal risk of complications and reduced economic burden.

One such marker free alternative is a narrow band imaging cystoscopy (NBI). NBI uses light at different wavelengths than WLC and allow to see hyper vascularized areas of the mucosa more clearly in contrast with healthy tissue. Studies that have compared NBI to WLC show, that NBI increases sensitivity, however, has a significantly lower specificity (50% vs. 86.9%, *p* < 0.001) and an increase in unnecessary biopsies [9].

A variety of optical spectroscopy methods including fluorescence, Raman scattering, and infrared (IR) absorption spectroscopy becomes more and more widely applied for the investigation of biological tissues in the field of surgical oncology. Methods of fluorescence are already applied for the surgical procedures. Fluorescence guided surgery is already being used for several types of oncological surgery [10]. During the surgery, this method allows to visualize different tissue types such as normal tissue and malignant lesions that would not be visible under the naked eye inspection. However, there are some limitations. In order to visualize different tissue types, fluorescence contrast agent should be inserted into the body before the investigation to be accumulated in the targeted tumorous tissue area. In this regard, vibrational spectroscopy methods (Raman scattering and IR absorption) have some advantages since they can be considered as a label-free methods without requirement of tissue dyeing or chemical processing prior the examination.

Methods of spontaneous and coherent Raman scattering spectroscopy were applied for the tumorous tissue identification in human and animal models and showed promising results [11]. In particular, applied fiber-based technique for the brain tissue examination showed the ability to perform the investigation under in vivo conditions and showed good results for the intraoperative tumor detection [12]. However, Raman spectroscopy has some limitations. Raman scattering is a relatively weak process therefore the signal of Raman scattering usually is not strong and particularly sensitive detectors should be used in order to receive a sufficient Raman signal. Moreover, depending on the wavelength and power of the laser that is used for the excitation of the Raman scattering, undesirable effects of fluorescence background in the Raman spectra can be observed. Attention to the surrounding light sources also should be paid: Radiation from the other light sources used in the operating room could interfere with the operating mode of the detector and have influence to the obtained spectra. Additionally, the usage of laser radiation in Raman spectroscopy can be problematic: the power of the laser and the integration time of the measurement should be carefully selected in order to avoid the damage of the tissue, and at the same time obtain the sufficient Raman signal and to receive the data in a reasonable time to maintain the examination time as short as possible without prolonging the surgery and anesthesia time.

Meanwhile, IR radiation is non-destructive for biological tissues and a spectrum can be obtained quite fast. IR spectroscopy is already used for studies of various biological samples including human tumorous tissue or cells [13,14,15,16,17,18,19]. Applied methods cover conventional techniques such as IR transmission, micro spectroscopy and attenuated total reflection (ATR). In most cases, conventional methods require a special sample preparation as well as placement of the samples to the special sample compartment inside the spectrometer. In addition, standard spectrometers usually are bulky and non-maneuverable that is an obstacle for the implementation of the method into the operating room. Application of fiber optics for the IR spectroscopy enables to bring the method one step forward clinical diagnostics. This provides the capability of performing the measurements of spectra under in situ conditions without the requirement to transfer the tissue into the device. Moreover, mobile devices of small dimensions could be coupled with fiber optics thus allowing to transfer the equipment almost anywhere in the operating room without the disturbing the flow of the surgery.

Some authors report results of application of IR spectroscopy for human BC investigation, however, most of them cover applications for specially prepared tissue samples such as formalin-fixed and paraffin-embedded tissues [20,21] or cultured cell lines fixed in glutaraldehyde in Phosphate-Buffered Saline (PBS) [22]. In our previous studies we showed that fiber-based IR spectroscopy can be successfully applied for examination of various freshly resected human tissues [23,24]. Here we represent the study of 54 patients by using fiber-based IR spectroscopy to delineate normal and tumorous human bladder tissues under ex vivo conditions. To our knowledge, in this manuscript we for the first time present the study of fiber probe application for freshly resected untreated human bladder tissue samples. Investigation of the tissue samples immediately after surgical operation allows to examine samples in their native conditions and establish their initial structure and chemical composition avoiding sample degradation. After further implementations, the method could be used even in vivo conditions thus allowing easier tissue examination during the surgery.

## 2. Materials and Methods

### 2.1. Sample Collection

Spectral studies of the bladder tissues were approved by the Regional Biomedical Research Ethics Committee (Document No. 2019/12-1178-665). The details of the sample collection methodic is described in our previous paper [25]. Briefly, the samples of the bladder tissues for fiber-based ATR IR spectroscopic studies were obtained between July 2019 and September 2021 in the tertiary Urology Center when performing TURB procedure. Patients were eligible if they had a clinical or radiological suspicion of BC, and they were required TURB procedure according to the latest NMIBC guidelines of the European Association of Urology.

Samples were collected during the TURB procedure in the following manner: we began by obtaining single sample of healthy-looking bladder tissue, then–single sample of the bladder superficial tumor. After the procedure, bladder tissue samples were sent for histological and spectroscopic analysis. Tumor malignancy was confirmed by a pathologist by examining the resected tissue.

### 2.2. Spectroscopy

IR spectra of freshly resected tissue samples were collected using ATR IR fiber probe. The system consists of the ATR silver halide fiber probe (Art Photonics GmbH, Berlin, Germany) attached to the standard FT-IR (Fourier transform infrared) spectrometer Alpha (Bruker Optik GmbH, Ettlingen, Germany) additionally equipped with the external liquid nitrogen cooled MCT (Mercury-Cadmium-Telluride) detector (Infrared Associates, Inc. Model IRA-20-00131).

Small cuts of normal and tumorous bladder tissue were made. Spectra collected by pressing the ATR fiber probe to the surface of freshly cut tissue. For each sample several spectra were measured and were averaged for the analysis. Spectra collected in the spectral region between 400 and 4000 cm^−1^ with 4 cm^−1^ spectral resolution. Sixty-four interferograms were averaged and Fourier transformed into a spectrum applying Blackmann-Harris 3 term apodization function and zero filling factor of 2. Before every measurement ATR crystal was cleaned with distilled water and ethanol and the background spectrum of the ambient air was measured.

### 2.3. Statistical Analysis

Statistical analysis (Hierarchical cluster analysis (HCA) and Principal component analysis (PCA)) was performed by using data analysis software OriginPro (OriginLab corporation). Prior statistical analysis, spectra were pre-processed: atmospheric compensation, baseline correction, vector normalization and offset correction were applied by using spectroscopy software OPUS (Bruker Optik GmbH, Ettlingen, Germany). Before performing HCA analysis, first derivation of spectra was calculated by using spectroscopy software OPUS. Statistical analysis was performed by applying standard procedures (Hierarchical Cluster Analysis and Principal Component Analysis) of the OriginPro software package. While performing HCA, Ward cluster algorithm, Euclidean distance type were chosen. For both statistical methods (HCA and PCA), analysis was performed in 950–1480 cm^−1^ spectral range. Different spectral regions were chosen for statistical analysis; however, the best results were achieved by performing analysis in the mentioned spectral range.

In order to evaluate the reliability of the method, sensitivity, specificity, positive predictive value (PPV) and negative predictive value (NPV) were calculated as follows:

$$Sensitivity=\frac{True\;positives}{Truepositives+Falsenegatives},$$

$$Specificity=\frac{True\;negatives}{Truenegatives+Falsepositives},$$

$$PPV=\frac{True\;positives}{Truepositives+Falsepositives},$$

$$NPV=\frac{True\;negatives}{Truenegatives+Falsenegatives}.$$

95% Confidence intervals (CI) were calculated by using MedCalc (MedCalc Software Ltd., Ostend, Belgium) online tool [26] for statistical analysis.

In total, 54 patient cases were analyzed. The summarized data is shown in the Table 1. In most cases, two types of tissue samples (normal and tumorous) were received for spectral analysis per patient. In several cases, only normal or only tumorous tissue was available for analysis from particular patient, thus only 49 normal and 47 tumorous tissue samples were analyzed. More detailed information is presented in Supplementary material (Table S1).

## 3. Results

**Figure 1:**
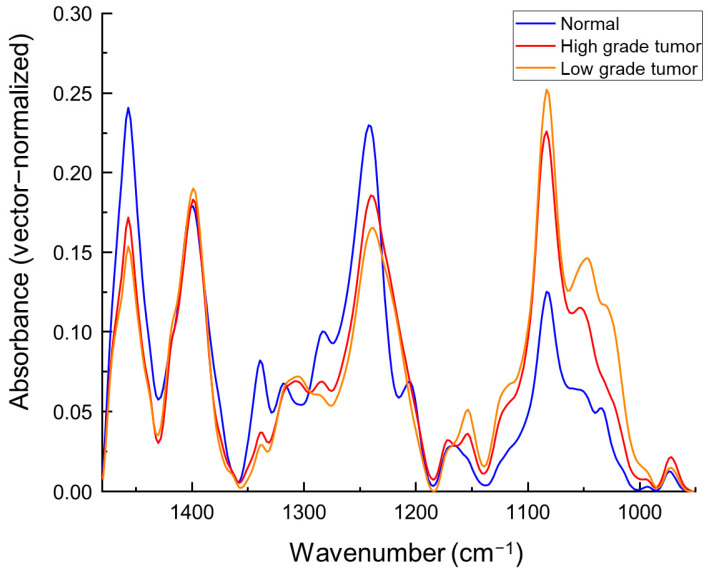
Mean ATR IR spectra of normal and urothelial carcinoma (high-grade tumor and low-grade tumor) tissue. Spectra are baseline corrected and vector normalized.

Mean ATR IR spectra of normal and tumorous bladder tissues presented in Figure 1 demonstrate obviously different profiles. Main spectral band positions and their assignments are shown in Table 2.

Mean spectrum of normal bladder tissue represents more prominent spectral bands located at 1033, 1206, 1240, 1282, 1317 and 1339 cm^−1^ which can be assigned to collagen [27,28] and indicates higher collagen levels in normal bladder tissue. Spectra of tumorous bladder tissue demonstrate higher absorbance values of the spectral band located at 972 cm^−1^ which could be assigned to ν(PO_4_) vibrations of nucleic acids. Moreover, spectral bands located at similar positions can be assigned to ν(PO_4_) vibrations of proteins and vibrational modes of OCH_3_ groups of polysaccharides. However, herewith the increased absorbance values of this spectral band, the stronger absorption is for the spectral band located at 1082 cm^−1^ in tumorous tissue spectra and assigned to ν(PO^−^_2_) vibrations of nucleic acids. These findings can be related to increased levels of nucleic acids in urothelial carcinoma tissue. Higher values of absorbance for the band positions at 1028 cm^−1^ assigned to ν(C-O), ν(C-C), δ(C-O-H) vibrations and at 1154 cm^−1^ assigned to ν(C-O) vibrations of glycogen are observed.

**Figure 2:**
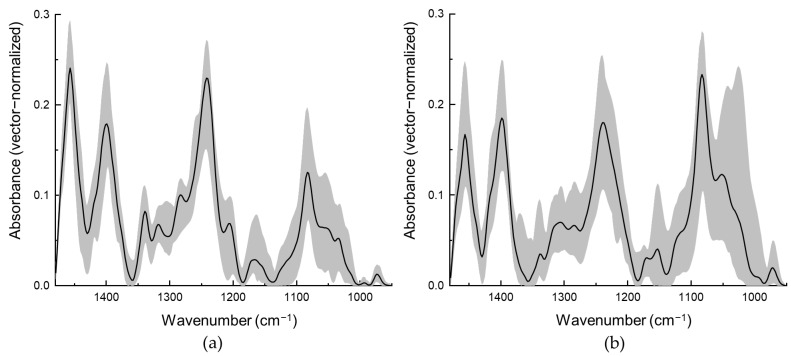
Mean spectra (black solid line) and variations of spectra of tissue taken from different patients (grey area) of normal (**a**) and tumorous urothelial carcinoma (**b**) tissue.

Although differences between the mean spectra of normal and tumorous tissues are clearly visible and well defined, variations of spectra inside both tissue classes (normal and tumorous tissues) are quite high (Figure 2).

Black lines correspond to the mean spectra of all the samples taken from different patients, while the grey areas indicate the variations of spectra in different tissue classes. Variations of spectra in the tumorous tissue class are higher (Figure 2b), especially for the spectral bands corresponding to glycogen (located at 1028 and 1154 cm^−1^) and collagen (1240, 1284, 1317, 1339 and 1457 cm^−1^) and could be considered as spectral tumor markers of urothelial carcinoma.

**Figure 3:**
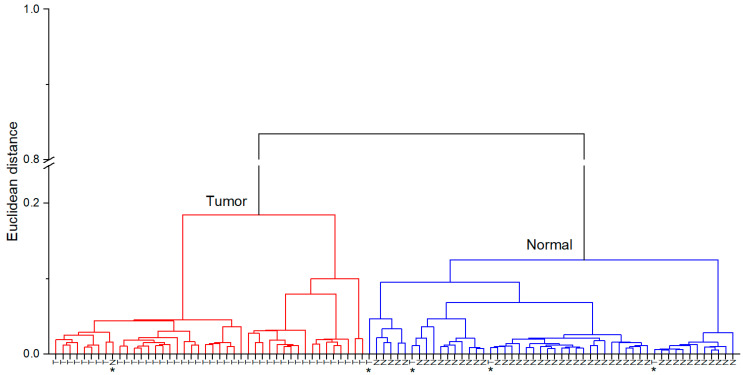
HCA dendrogram. Red cluster corresponds to the tumorous tissue class, blue cluster corresponds to normal tissue class. Letters T and N represent spectra of each tumorous and normal tissue samples, respectively. * Symbol indicates spectra which were assigned to wrong tissue class.

In order to evaluate the capability of the IR spectroscopy to delineate tumorous and normal tissues, HCA and PCA analysis were applied. Figure 3 shows the dendrogram of HCA analysis. Spectra of tumorous and normal tissues are classified into 2 clusters (red cluster on the left and blue cluster on the right side of the dendrogram correspond to tumorous and normal tissue classes, respectively), large distance between clusters reveals a good separation between both tissue classes. Only 1 spectrum out of 49 normal tissue spectra and 4 spectra out of 47 tumorous tissue spectra were assigned to wrong classes (these spectra are indicated by * symbol) that corresponds to 91% sensitivity and 98% specificity (Table 3).

**Figure 4:**
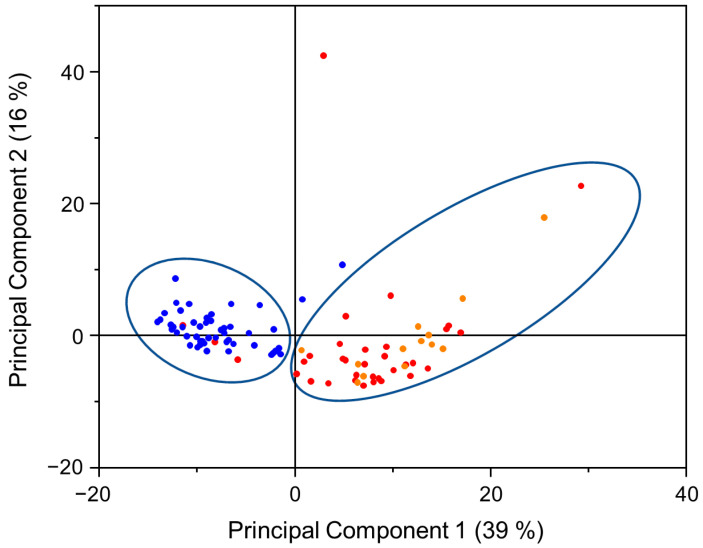
PCA diagram. Blue dots indicate spectra of each normal tissue samples, red and orange dots indicate spectra of each tumorous (high- and low-grade of malignancy, respectively) tissue samples. Percentage of variance for PCs is shown in brackets.

Results of PCA analysis are presented in Figure 4. Spectra are normalized thus the most part of variance is explained by first and second principal components (PCs) (percentage of variance is 39% and 16% for the first and second PCs, respectively). The most significant chemical changes in the tissue samples are defined by first and second PCs, thus the score plot is shown for these two PCs. Clusters on the left and right sides of the diagram correspond to normal and tumorous tissue classes, respectively. Blue dots indicate spectra of each normal tissue, red and orange dots indicate spectra of each tumorous (high- and low-grade of malignancy, respectively) tissue samples. Classes are well separated, only 2 spectra out of 49 normal tissue spectra and 4 spectra out of 47 tumorous tissue spectra were assigned to wrong classes that corresponds to 91% sensitivity and 96% specificity (Table 3).

## 4. Discussion

As it is observed from the ATR IR spectra (Figure 1), normal bladder tissues are richer in collagen compared to urothelial carcinoma tissues. Collagen is one of the components of the lamina propria of urinary bladder and assures the tensile strength by transferring the tension from the bladder smooth muscle cells, during the expansion of the organ while storing urine [29]. Reduced collagen levels in tumorous bladder tissue can be related to increased enzymes secretion of collagenase into the tissue matrix and destruction of host tissue [30]. Possibly higher levels of nucleic acids in urothelial carcinoma tissue can be related to higher rates of cell proliferation due to growth of the tumor. Increased absorbance values of spectral bands assigned to glycogen are related to increased amounts of glycogen in tumorous bladder tissues. Increased levels of glycogen are observed in some types of tumors and used as the resource of energy for the cell proliferation [31,32]. Moreover, higher levels of glycogen are observed in low-grade urothelial carcinoma tissue compared to high-grade tumor tissue. Observed inverse relation of glycogen amount and tumor grade could be related to higher proliferation rates of high-grade tumors. In some types of tumors inverse relation of glycogen amount and proliferation rates are observed and possibly linked to higher glycogen consumption for maintaining tumor growth [32].

Higher variations in tumorous tissue spectra class compared to normal tissue spectra class could be linked to the diverse differentiation of tumorous cells. During development of the tumor, cells lose their functions and change their morphological features. Thus, depending on the tumor features and development, variations of the amounts of specific components can vary in individual cases. Meanwhile variations in normal tissue spectra class are smaller (Figure 2a) compared to tumorous tissue class. Spectral differences may be caused by individual differences in tissue constitution.

In this study, presented sensitivity values reach 91% (95% CI 80–98%) (by applying both HCA and PCA methods) while specificity values reach 98% (95% CI 89–100%) and 96% (95% CI 86–100%) by applying HCA and PCA methods, respectively. Currently, most widely used endoscopic approach to diagnose BC is WLC with sensitivity of 71% (95% CI, 49–93%) and specificity of 72% (95% CI, 47–96%) [33]. A novel technique with instillation of photosensitizing agent, so called photodynamic diagnosis (PDD), has shown better sensitivity rates of (92% (95% CI, 80–100%)) but lower specificity (57% (95% CI, 36–79%)) [33]. Another novel technique known as NBI is based on less invasive process of bladder wall illumination with filtered white light. It enhances the visualization of mucosal blood vessels because emitted wavelengths are absorbed by hemoglobin more intensively compared to other mucosal tissue and helps to contrast the neoangiogenic urothelial tumors [34]. According to the literature, NBI offers high sensitivity (96% (95% CI = 93–98%)) but low specificity (65% (95% CI = 54–75%)), similarly to PDD [5].

Our study has showed promising results to differentiate healthy and cancerous tissue of urinary bladder under ex vivo conditions, which are close to the golden standard of pathological examination. Fiber-based ATR IR spectroscopy showed similar sensitivity results as the results in the literature of PDD and NBI without a decrease in specificity. This could lead to less invasive diagnostic approach, when correct diagnosis would not require excessive resection of the bladder. Moreover, high specificity of the test could lead to a decrease in numbers of pathological analyses required in the follow-up of the patient with reduced economic burden.

However, our study shows some limitations. Firstly, we were unable to obtain both cancerous and normal bladder tissue samples from every patient. Surgeries were performed for patients with suspected malignant bladder neoplasms. Resected tissue was evaluated macroscopically by a surgeon; subsequently, suspicious looking tissue was assigned as tumorous and a sample of resected tissue was given for spectral analysis, the whole resected tissue was sent for routine pathological examination. After pathological examination, 6 samples suspected as tumorous finally were identified as chronic cystitis. Dendrogram of HCA analysis including these 6 cystitis cases is presented in supplementary material (Figure S1). In one case it was impossible to obtain tumorous tissue. These 7 patient cases were not included in the statistical analysis presented in the main text of this paper. Other patients were diagnosed with completely altered bladder and we were unable to obtain normal bladder tissue samples. In this case, 5 patients were improper to obtain normal bladder tissue. Secondly, despite observed differences in mean spectra of low-grade and high-grade tumors, spectral profiles of low- and high-grade cancerous tissue share similar features thus not allowing to discriminate them into separate groups. This could be explained by different number of low- and high-grade tumor cases included in the study. Number of high-grade tumor cases is much bigger than low-grade tumor cases (34 and 13 cases, respectively), thus for the more detailed conclusions a larger scope of low-grade tumor cases would be beneficial. However, observed spectral features and applied statistical analysis allow to discriminate urothelial carcinoma and normal bladder tissues with high accuracy rates under ex vivo conditions on cystoscopically selected tissue samples. Based on our study data, fiber-based ATR IR spectroscopy could be an addition to current endoscopic approach with an increase in diagnostic accuracy, if successfully applied in vivo.

## 5. Conclusions

This study shows the possible ex vivo application of the fiber-based ATR IR spectroscopy for the discrimination between the cancerous and normal human bladder tissues with promising results for further studies. High rates of tissue identification accuracy suggest that the applied method after some implementations could be used in addition to current diagnostic approaches.

## Figures and Tables

**Table 1:** Tissue types collected and patient characteristics during the study. Information based on histological analysis, surgery information.

Tissue Type	Number of Samples
Normal tissue	49
Tumorous tissue	
High-grade tumor	34
Low-grade tumor	13
Patient characteristics	Number of patients
Tumor size during initial TURB	
<10 mm	12
10–30 mm	18
>30 mm	17
Tumor count on initial TURB	
Single	15
Two to three	15
More than three	17
Additional information	
Number of patients Median patient age (years)	54 70.5

**Table 2:** Spectral bands and their assignments [27].

Spectral Band Position, cm^−1^		Assignment
Normal Tissue	Urothelial Carcinoma Tissue	
972	972	ν(PO_4_) of nucleic acids and proteins OCH_3_ of polysaccharides
Not present	1028	ν(C-O), ν(C-C), δ(C-O-H) of glycogen
1033	Not present	Collagen
1050	1048 (low-grade) 1052 (high-grade)	ν(CO-O-C) ν(C-O), δ(C-O) of the C-OH of carbohydrates
1082	1082	ν(PO^−^_2_) of nucleic acids
Shoulder at 1121	Shoulder at 1121	ν(C-O), δ(C-O), δ(C-O-H), δ(C-O-C) phosphodiester stretching
Shoulder at 1154	1154	ν(C-O)
1163	Not present	ν(C-O)
Shoulder at 1172	1172	ν(C-O)
1206	Not present	Amide III
1241	1239	ν(PO^−^_2_), Amide III
1282	1284	Amide III
Not present	1307	ν(CH_2_)
1317	Shoulder at 1317	Amide III
1339	1339	CH_2_ wagging
1399	1399	δ(CH_3_)
1457	1457	δ(CH_3_)

**Table 3:** Summarized values of sensitivity, specificity, PPV and NPV values for HCA and PCA results.

	Method			
	HCA		PCA	
	Value	95% CI	Value	95% CI
Sensitivity	91%	80% to 98%	91%	80% to 98%
Specificity	98%	89% to 100%	96%	86% to 100%
PPV	98%	86% to 100%	96%	85% to 99%
NPV	92%	82% to 97%	92%	87% to 98%

CI–confidence interval.

## Data Availability

The data can be shared up on request.

## Supplementary Materials

The following supporting information can be downloaded at: https://www.mdpi.com/article/10.3390/cancers15020499/s1. Table S1: Detailed patient and tissue sample information. Figure S1: HCA dendrogram of benign and malignant tissues.
