# Supplementary material for: Differentiation of Urothelial Carcinoma and Normal Bladder Tissues by Means of Fiber-Based ATR IR Spectroscopy
Source: Cancers (Basel). 2023 Jan 13;15(2):499. doi: 10.3390/cancers15020499 (PMC9857111; doi:10.3390/cancers15020499)
Supplement: Supplementary file 1 [file cancers-15-00499-s001.zip › cancers-2023544-supplementary.pdf]

Supplementary material

Differentiation of Urothelial Carcinoma and Normal Bladder Tissues by Means of Fiber-Based ATR IR Spectroscopy

Rimantė Bandzevičiūtė [1,*], Gediminas Platkevičius [2,*], Justinas Čeponkus [1], Arūnas Želvys [2], Albertas Čekauskas [2] and Valdas Šablinskas [1]

[1] Institute of Chemical Physics, Faculty of Physics, Vilnius University, Saulėtekio av. 3, LT-10257 Vilnius, Lithuania

[2] Institute of Clinical Medicine, Faculty of Medicine, Vilnius University, M. K. Čiurlionio g. 21/27, LT-03101 Vilnius, Lithuania

* Correspondence: rimante.bandzeviciute@ff.vu.lt (R.B.); gediminas.platkevicius@santa.lt (G.P.)

Table S1. Detailed patient and tissue sample information.

Patient	Tissue type received		Diagnosis
	Normal	Pathological	
Patient01	+	N/A	High-grade urothelial carcinoma
Patient02	+	+	High-grade urothelial carcinoma
Patient04	+	N/I	Chronic cystitis
Patient05	+	+	High-grade urothelial carcinoma
Patient06	+	+	High-grade urothelial carcinoma
Patient07	+	+	Low-grade urothelial carcinoma
Patient08	+	+	High-grade urothelial carcinoma
Patient09	+	+	Low-grade urothelial carcinoma
Patient10	+	N/I	Chronic cystitis
Patient11	+	+	Low-grade urothelial carcinoma
Patient12	+	+	High-grade urothelial carcinoma
Patient13	+	N/I	Chronic cystitis
Patient14	+	+	High-grade urothelial carcinoma
Patient15	+	+	High-grade urothelial carcinoma
Patient16	+	+	High-grade urothelial carcinoma
Patient17	+	+	High-grade urothelial carcinoma
Patient18	+	+	High-grade urothelial carcinoma
Patient19	+	+	High-grade urothelial carcinoma
Patient20	+	+	Low-grade urothelial carcinoma
Patient21	+	+	High-grade urothelial carcinoma
Patient22	+	+	High-grade urothelial carcinoma
Patient23	+	+	High-grade urothelial carcinoma
Patient24	N/A	+	High-grade urothelial carcinoma
Patient25	+	+	Low-grade urothelial carcinoma
Patient26	+	N/I	Chronic cystitis
Patient27	+	+	High-grade urothelial carcinoma
Patient28	+	+	Low-grade urothelial carcinoma
Patient29	+	+	High-grade urothelial carcinoma
Patient30	+	N/I	Chronic cystitis
Patient31	N/A	+	High-grade urothelial carcinoma
Patient32	+	+	High-grade urothelial carcinoma
Patient33	+	+	Low-grade urothelial carcinoma
Patient34	+	+	High-grade urothelial carcinoma
Patient35	+	+	High-grade urothelial carcinoma
Patient36	+	+	Low-grade urothelial carcinoma
Patient37	N/A	+	High-grade urothelial carcinoma
Patient38	+	+	Low-grade urothelial carcinoma
Patient39	+	+	High-grade urothelial carcinoma
Patient40	+	+	High-grade urothelial carcinoma
Patient41	N/A	+	High-grade urothelial carcinoma
Patient42	+	+	High-grade urothelial carcinoma
Patient43	+	+	High-grade urothelial carcinoma
Patient44	+	+	High-grade urothelial carcinoma
Patient45	+	+	Low-grade urothelial carcinoma
Patient46	+	N/I	Chronic cystitis
Patient47	N/A	+	High-grade urothelial carcinoma
Patient48	+	+	High-grade urothelial carcinoma
Patient49	+	+	Low-grade urothelial carcinoma

Patient50	+	+	High-grade urothelial carcinoma
Patient51	+	+	High-grade urothelial carcinoma
Patient52	+	+	Low-grade urothelial carcinoma
Patient53	+	+	Low-grade urothelial carcinoma
Patient54	+	+	High-grade urothelial carcinoma

+ - tissue sample received, N/A - not available, N/I - not included in the main statistical analysis

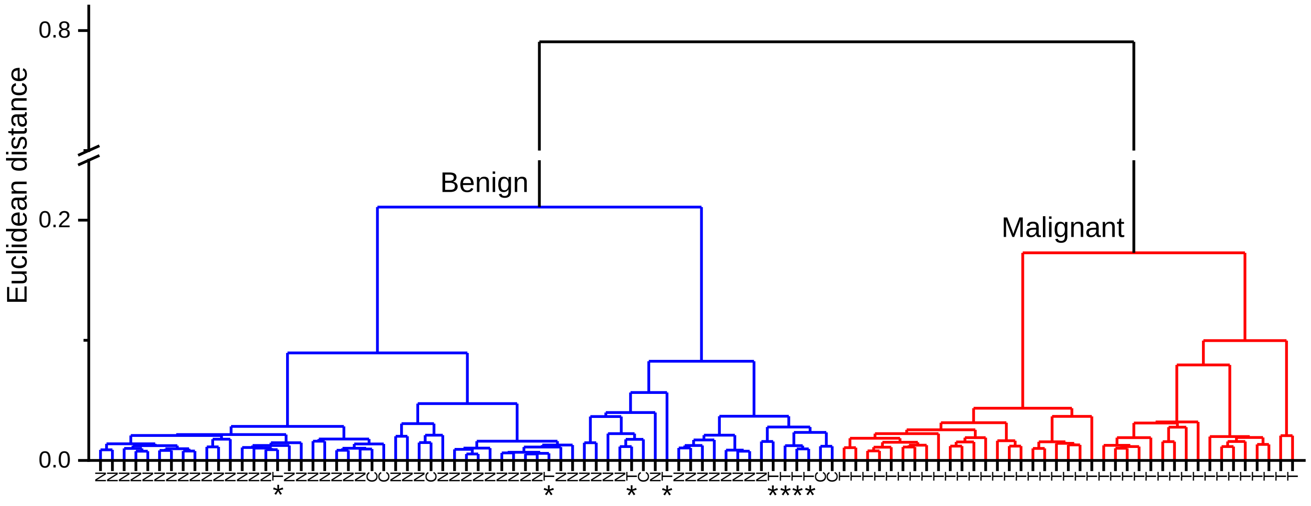

Figure S1. HCA dendrogram of benign and malignant tissues. After application of HCA analysis for all resected tissue samples, including normal, tumorous and cystitis tissues, data are classified into two well separated clusters which could be referred as benign and malignant classes. Blue cluster corresponds to benign tissue class including normal and cystitis tissues, red cluster corresponds to malignant tissue class including urothelial carcinoma tissues. Letters N, T and C represent spectra of normal, tumorous and cystitis tissue samples respectively. * symbol indicates tumorous tissue spectra which were assigned to benign tissue class. As it is observed from the graph, spectra of all normal and cystitis tissue samples are grouped into one cluster labelled as benign tissue class, meanwhile 8 spectra of urothelial carcinoma tissue are also classified as benign tissue. The identification accuracy of benign and malignant tissues is 100 % and 83 % respectively. Obtained tissue identification accuracy is lower compared to the accuracy when only normal and urothelial carcinoma tissues are analysed. This could be determined by small number of cystitis tissue samples. For more accurate classification, more samples of cystitis tissue are required. Statistically comparable amounts of three classes of samples probably will lead to the possibility to separate them in to three individual clusters.
